# Association Between Mandibular Anterior Crowding and Gingival Recession Following Orthodontic Treatment: A Retrospective Cohort Study

**DOI:** 10.3390/dj14080498

**Published:** 2026-08-07

**Authors:** Sarah Fita, Tracey Whitley, Robin Henderson, Onur Kadioglu, Steven Pan

**Affiliations:** 1 Department of Preventive Dental Sciences, Imam Abdulrahman bin Faisal University, Dammam 34221, Saudi Arabia; 2 Division of Periodontics, Department of Diagnostic and Preventive Sciences, College of Dentistry, University of Oklahoma Health Sciences, Oklahoma City, OK 73117, USA; tracey-whitley@ou.edu (T.W.); robin-henderson@ou.edu (R.H.); 3 Division of Orthodontics, Department of Developmental Dentistry, College of Dentistry, University of Oklahoma Health Sciences, Oklahoma City, OK 73117, USA; onur-kadioglu@ou.edu; 4 Department of Biostatistics and Epidemiology, Hudson College of Public Health, University of Oklahoma Health Sciences Center, Oklahoma City, OK 73117, USA; steven-pan@ou.edu

**Keywords:** mandibular crowding, gingival recession, oral hygiene, orthodontic treatment

## Abstract

**Background/Objectives:** Gingival recession frequently affects the anterior mandibular region. Anatomical features, such as thin buccal bone, narrow keratinized gingiva, and root proximity, increase susceptibility to periodontal disease. Recession can progress over time and may be exacerbated by orthodontic treatment. However, it remains uncertain whether the severity of crowding before treatment contributes to recession after orthodontic therapy The present study aimed to determine whether a relationship exists between the degree of mandibular anterior crowding before orthodontic treatment and the development or progression of gingival recession after treatment completion. **Methods:** This retrospective study was conducted on 269 participants with pre- and post-treatment records. The Little’s irregularity index was used to assess the degree of crowding. Clinical photographs of the six mandibular anterior teeth (1614 sites) were digitally calibrated permit millimeter-based assessment of gingival recession and keratinized tissue (KT) width. Oral hygiene (OH) was also evaluated at both time points. **Results:** Post-treatment recession was detected in 76 teeth. (4.7% of sites) among 48 participants (17.8%). Pretreatment crowding severity was not significantly associated with the occurrence of recession after treatment. Pretreatment mucogingival deformities were independently associated with post-treatment gingival recession. Mandibular central incisors demonstrated the highest prevalence of post-treatment gingival recession. **Conclusions:** Pretreatment mandibular anterior did not appear to predict gingival recession at treatment completion. Periodontal characteristics may play a more important role in recession development, supporting careful periodontal evaluation before and throughout orthodontic care.

## 1. Introduction

Gingival recession is a common mucogingival condition that has esthetic and functional effects, including dentin hypersensitivity, pain, plaque retention, gingival bleeding, and esthetic concerns [1]. This multifactorial condition has been associated with anatomical, physiological, and pathological factors [2]. The relationship between orthodontic treatment and gingival recession has been widely investigated, yet findings remain inconclusive [3]. Notably, the mandibular anterior region has been reported as the most frequently affected site for gingival recession following orthodontic treatment [4]. Understanding factors that influence recession risk is therefore clinically relevant for treatment planning and risk assessment.

Mandibular anterior teeth present with several anatomical characteristics that may predispose them to periodontal disease, including dental crowding [5], root proximity [6], thin bone morphotype [7], thin gingival phenotype [8], and a higher prevalence of buccal fenestrations and dehiscence defects [9]. These features have been associated with various forms of periodontal breakdown [10,11,12]. Incisor crowding has been linked to periodontal disease progression, manifested by increased probing depths and alveolar bone loss [10]. Root proximity, defined as an interradicular distance of ≤0.8 mm at the level of the cementoenamel junction (CEJ), has been shown to exhibit a dose-dependent association with alveolar bone loss, whereas greater distances appear to confer minimal risk [11]. Additionally, a thin gingival phenotype (<1 mm), particularly in the coronal third of the periodontal tissues, has been associated with an increased likelihood of subsequent gingival recession [8].

Previous studies have investigated the development of gingival recession following orthodontic treatment either at the end of treatment or during short- and long-term follow-up periods. The highest prevalence of labial recession has been reported in mandibular central incisors immediately after appliance removal and during retention [13,14]. While some investigations have identified orthodontic variables, such as incisor proclination, as potential contributors to recession development [15,16], others have emphasized periodontal characteristics including gingival phenotype, keratinized tissue width, and pre-existing inflammation [16,17]. However, the influence of pretreatment mandibular anterior crowding has not been consistently evaluated. Despite crowding being associated with periodontal disease indicators such as pocket depth and alveolar bone loss [10], its independent contribution to gingival recession following orthodontic treatment remains unclear. Therefore, this study investigated whether the severity of pretreatment mandibular anterior crowding was associated with the development or progression of gingival recession by the end of orthodontic treatment.

## 2. Materials and Methods

### 2.1. Study Design and Ethical Approval

Using a retrospective clinical design, the study examined, the association between mandibular anterior crowding and the incidence or progression of gingival recession during orthodontic treatment. The study sample comprised individuals who received care at the Graduate Orthodontics Clinic of the University of Oklahoma Health Sciences Center (OUHSC) between 2007 and 2022. Institutional Review Board approval was obtained (IRB #15019), and the study complied with the Health Insurance Portability and Accountability Act. The study was conducted and reported in accordance with the Strengthening the Reporting of Observational Studies in Epidemiology (STROBE) guidelines. Patients aged 12–18 years who had completed orthodontic treatment and had clinical records from both before and after treatment available at the clinic were identified from the University of Oklahoma College of Dentistry’s existing database. All records were deidentified, and patients were assigned sequential identification numbers.

### 2.2. Sample Selection

The inclusion criteria included patients aged 12–18 years who had fully erupted permanent teeth extending between the first molars in each arch at the start of treatment, controlled systemic health, and availability of complete pre-treatment and post-treatment clinical records. Records before the start of orthodontic therapy included intraoral photographs and diagnostic casts, whereas post-treatment records included intraoral photographs taken after orthodontic appliance removal. The exclusion criteria included patients aged <12 years or >18 years, with a history of smoking, systemic conditions affecting the periodontium (such as diabetes and osteoporosis), craniofacial anomalies, mixed dentition, premolar extraction cases, previous orthognathic, periodontal, or mucogingival surgery, orthodontic treatment performed in combination with surgery, or restorations involving the mandibular anterior region near or below the gingival margin. This study represents a secondary analysis of a larger retrospective cohort, from which premolar extraction cases were excluded to create a more homogeneous sample.

### 2.3. Assessment of Mandibular Anterior Crowding

Crowding severity was assigned to one of five categories using Little’s irregularity index, resulting in five groups [18]. This index quantifies the mandibular anterior misalignment by measuring the linear displacement between the anatomic contact points of the six anterior teeth. Digital models were generated from scanned mandibular study casts using a 3Shape Trios 3 intraoral scanner (3Shape A/S, Copenhagen, Denmark), and the resulting Standard Tessellation Language (STL) files were imported into the three-dimensional (3D) Slicer software (version 5.2.1; Slicer Community) for measurements. Five measurements were taken at adjacent contact points using the linear ruler tool, and their sum was the total irregularity score. Based on these values, the patients were classified into five categories: 0 mm, perfect alignment (Group 1); 1–3 mm, minimal irregularity (Group 2); 4–6 mm, moderate irregularity (Group 3); 7–10 mm, severe irregularity (Group 4); and >10 mm, very severe irregularity (Group 5). An additional classification was based on the total crowding, incorporating the sum of the arch-tooth size discrepancy and the curve of Spee correction values.

### 2.4. Clinical Measurements

Gingival recession and mucogingival deformities were evaluated using calibrated intraoral photographs obtained before and immediately after appliance removal. Calibration was performed in ImageJ (version 1.53u; U.S. National Institutes of Health, Bethesda, MD, USA) by referencing known measurements from pre-treatment dental casts, enabling accurate conversion of pixel dimensions to millimeters. This method was selected to allow standardized, consistent measurements across all participants in this retrospective study. All intraoral photographs were obtained as part of standardized orthodontic clinical records using consistent photographic protocols. Recession was visually detected by identifying the exposed root surfaces and measured at the buccal midpoint of each mandibular anterior tooth. The distance from the CEJ to the apical-most level of the gingival margin was recorded in millimeters. Mucogingival deformities recorded included lack of keratinized tissue (KT), high frenum attachment, and gingival recession. The width of KT was measured at the mid-buccal point as the distance from the gingival margin to the mucogingival junction (MGJ) and categorized into two groups: lack of KT (<2 mm) and adequate zone (≥2 mm), based on standard criteria. Aberrant/high frenum attachment was defined based on visual insertion level, with papillary and papilla-penetrating insertions classified as aberrant [19]. One calibrated examiner completed all measurements. Intra-examiner reliability was evaluated by repeating the measurements in a randomly selected subset after a two-week interval under identical conditions.

### 2.5. Oral Hygiene Assessment

The OH status was assessed pre- and post-treatment using clinical photographs and classified based on the system proposed by Julien et al. [20]. This visual assessment approach was used to maintain consistency with the available retrospective records and has been previously applied in orthodontic populations. At baseline, good OH was defined as no visible plaque or signs of gingivitis, fair OH included some plaque and localized gingivitis, and poor OH involved generalized thick plaque and inflammation. After treatment OH was considered good when plaque and gingival hypertrophy were absent and any bleeding was limited to areas of composite removal. Fair OH indicated visible plaque with localized gingivitis or hypertrophy and bleeding related to composite removal. Poor OH indicated plaque at multiple sites accompanied by generalized hypertrophy, gingivitis, and bleeding.

### 2.6. Statistical Analysis

**Figure 1:**
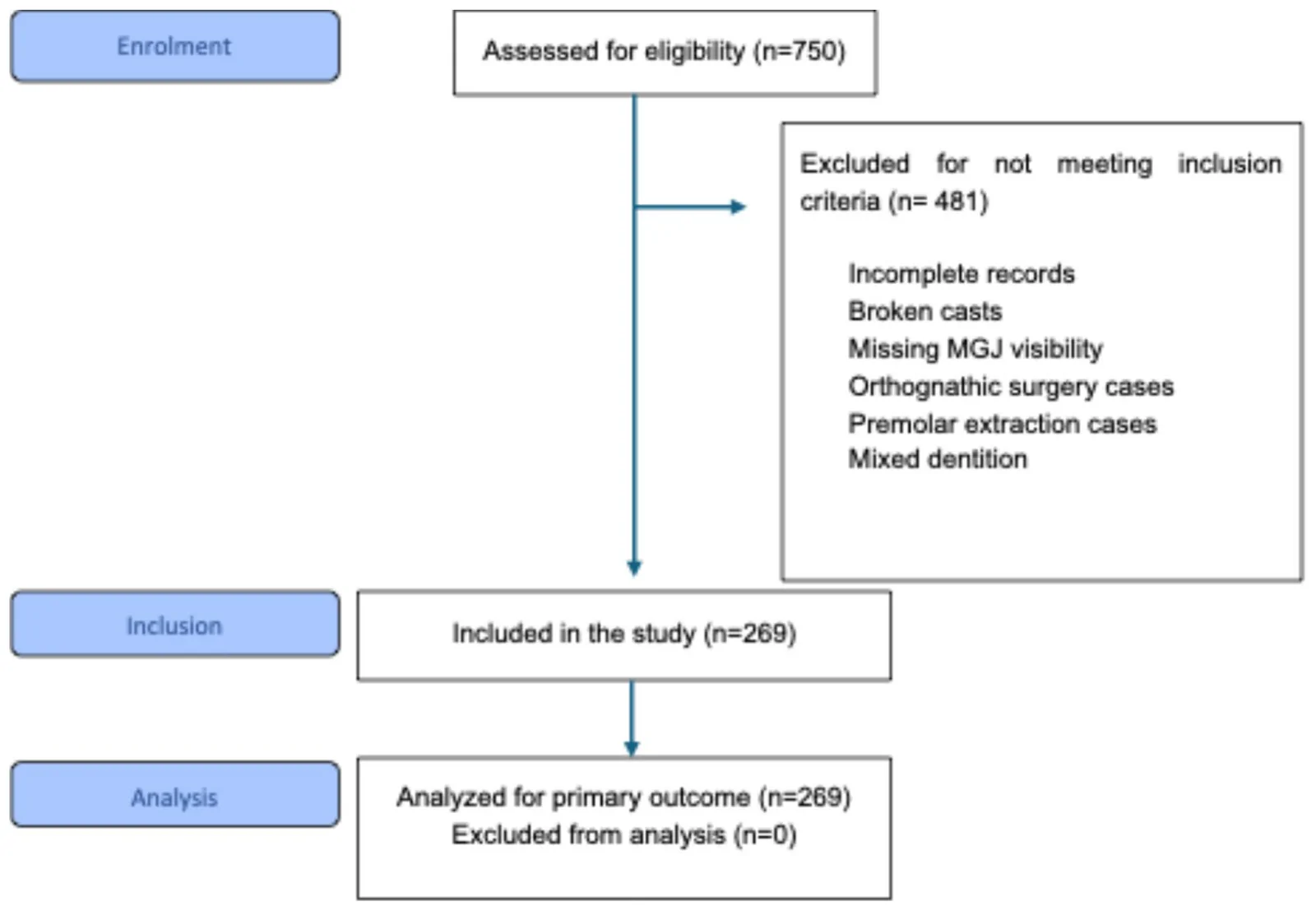
Flow diagram of participant selection according to STROBE guidelines.

Approximately 750 patient records were screened, of which 269 met the eligibility criteria and were included in the final analysis (Figure 1). The primary outcome was the presence of post-treatment gingival recession assessed at the subject level, while pretreatment mandibular anterior crowding was considered the primary exposure variable. Post-treatment gingival recession was defined at the patient level as the presence of recession (>0 mm) on at least one mandibular anterior tooth at the post-treatment assessment.

Continuous variables were summarized using medians and interquartile ranges (IQRs), and categorical variables were summarized using frequencies and percentages. Univariate comparisons between subjects with and without gingival recession were performed using the Wilcoxon rank-sum test for continuous variables and Pearson’s chi-square test or Fisher’s exact test for categorical variables, as appropriate. Secondary descriptive analyses were performed at the tooth level to evaluate recession distribution patterns among mandibular anterior teeth.

An additional multivariable logistic regression model was fitted to assess the relationship between pretreatment mandibular anterior crowding and post-treatment gingival recession after adjustment for potential confounding variables. The dependent variable was the presence or absence of post-treatment gingival recession. Pretreatment crowding severity was combined into three clinically relevant groups (perfect/mild, moderate, and severe/very severe) to reduce subgroup imbalance and improve statistical stability. The model was adjusted for age, sex, post-treatment oral hygiene status, and pretreatment mucogingival deformities. Post-treatment oral hygiene status was dichotomized as Good versus Fair/Poor for multivariable analysis. Adjusted odds ratios (ORs) with 95% confidence intervals (CIs) were calculated.

When multiple comparisons were performed, *p*-values were corrected using the false discovery rate method. Statistical significance was established at *p* < 0.05. Records with incomplete data were excluded during the selection process; therefore, no imputation methods were required.

## 3. Results

A total of 269 participants contributed 1614 sites (six per participant) for analysis in the present study. These were selected after screening approximately 750 orthodontic records according to eligibility criteria. The main reasons for exclusion were incomplete records, damaged casts, inadequate photographic visibility of the mucogingival junction, orthognathic surgery cases, mixed dentition, and premolar extraction cases. Table 1 summarizes the demographic and clinical profile of the sample.

Before the start of orthodontic therapy, none of the sample had high frenal attachment, 14 participants had gingival recession across 27 teeth, and 185 (68.8%) participants had at least one site with KT < 2 mm. Pretreatment mucogingival clinical parameters by tooth are presented in Table 2. Lack of keratinized tissue (KT < 2 mm) was observed in approximately half of the central incisors and canines and in 38–42% of the lateral incisors, and its distribution differed significantly according to tooth type (*p* < 0.001). Pairwise comparisons indicated that central incisors and canines demonstrated a greater lack of keratinized tissue compared with lateral incisors, whereas central incisors and canines did not differ significantly. Pretreatment gingival recession was uncommon, with no significant differences between teeth (*p* = 0.6).

At the end of orthodontic therapy, recession developed in 76 teeth (4.7% of sites) among 48 participants (17.8%). Table 3 illustrates the distribution of post-treatment gingival recession across the six mandibular anterior teeth. Central incisors demonstrated the highest prevalence (7.4–8.9%), followed by the canines (4.5–5.2%), whereas lateral incisors demonstrated the lowest prevalence (0.7–1.5%). The distribution differed significantly among tooth types (*p* < 0.001). Overall, mandibular central incisors demonstrated the highest burden of recession after treatment among the mandibular anterior teeth.

Table 4 shows a comparison of participants who developed gingival recession with those who did not. Age, malocclusion classification, crowding group, and degree of crowding were not significantly associated with recession development (*p* > 0.05). In univariate analyses, post-treatment oral hygiene demonstrated a significant association with recession (*p* = 0.043), with fair oral hygiene more frequently observed among participants who developed recession, whereas good OH was more common among those without recession.

After adjustment for age, sex, post-treatment oral hygiene, and pretreatment mucogingival deformity, pretreatment mandibular anterior crowding severity was not independently associated with post-treatment gingival recession (Table 5). The only variable independently associated with post-treatment gingival recession was the presence of a pretreatment mucogingival deformity (aOR 2.57, 95% CI 1.13–5.83, *p* = 0.024). Participants with pretreatment mucogingival deformities had 2.57 times greater odds of developing post-treatment gingival recession compared with those without pretreatment mucogingival deformities. Age, female sex, and fair/poor post-treatment oral hygiene were not independently associated with the outcome following multivariable adjustment.

## 4. Discussion

The present study found no relationship linking pretreatment crowding of the mandibular anterior teeth with post-treatment gingival recession. In contrast, pretreatment mucogingival deformities were independently associated with recession development. Gingival recession developed in 76 teeth (4.7% of sites) among 48 participants (17.8%), indicating a relatively low prevalence after orthodontic treatment.

The relatively low prevalence of gingival recession reported in this study is similar to findings reported in investigations looking at periodontal conditions after orthodontic treatment. Morris et al. reported a recession prevalence of approximately 5.8% at the completion of orthodontic therapy [14], whereas Vasconcelos et al. observed a prevalence of 10.3% in mandibular incisors shortly after treatment [13]. Additionally, Abdelhafez et al. compared recession prevalence following orthodontic treatment with an untreated control group and found slightly higher gingival recession development after treatment, but this difference was not statistically significant [21]. Variations in reported prevalence may partly reflect variations in follow-up periods, as several studies have shown that gingival recession tends to increase during the retention phase [14,16].

Assessing the site distribution of gingival recession revealed that mandibular central incisors were affected most frequently by recession at the end of orthodontic treatment, followed by the canines and lateral incisors. This pattern is consistent with previous studies reporting a greater susceptibility of mandibular central incisors to recession following orthodontic treatment [13,14]. The increased susceptibility of the central incisors may be related to anatomical characteristics of the mandibular anterior region, including thin buccal bone plates, root prominence, and limited keratinized tissue.

Although post-treatment oral hygiene demonstrated a significant association with recession in univariate analyses, this relationship did not persist following multivariable adjustment. Collectively, these findings support the multifactorial nature of gingival recession and suggest that baseline periodontal characteristics may have a greater influence on recession development than the severity of pretreatment crowding.

Literature on the interaction between orthodontic treatment and gingival recession has assessed multiple factors that can be broadly classified into orthodontic and periodontal factors. Several orthodontic variables, including malocclusion classification [17], lack of space [17], overjet [17], overbite [17], and proclination [17,22], have not consistently demonstrated an association with recession development. In contrast, periodontal characteristics such as thin gingival phenotype [17,23], inflammation [17], and reduced width of keratinized tissue [17], have been identified as potential risk indicators. However, the evidence remains heterogeneous on the effect of orthodontic and periodontal factors on gingival recession after orthodontic treatment. Yared et al. reported no association between plaque and gingival bleeding indexes, probing pocket depth with gingival recession [15], and a significant correlation between inclination > 95 degrees and free gingival margin thickness with gingival recession [15]. Gul reported overall similar findings to our study and the body of literature where periodontal parameters such as gingival phenotype, bleeding on probing, as well as age, were significantly associated with gingival recession, but not orthodontic tooth movement parameters [24].

Interestingly, Gül et al. also found that gingival phenotype, rather than keratinized tissue width, was associated with gingival recession [24]. This observation may reflect the multiple components of periodontal phenotype, which include gingival thickness, keratinized tissue width, and underlying bone morphotype [25,26]. Therefore, individual components such as keratinized tissue width may not fully capture the overall influence of periodontal phenotype on recession susceptibility.

The findings of the present study are generally consistent with this body of evidence. Although pretreatment mandibular anterior crowding was not independently associated with post-treatment gingival recession, pretreatment mucogingival deformities remained independently associated with recession development. Furthermore, although mucogingival deformities were evaluated as a composite variable, pretreatment recession was relatively uncommon (14 participants) and largely overlapped with participants exhibiting reduced keratinized tissue. Furthermore, no participants demonstrated high frenum attachment. Therefore, the observed association was likely driven primarily by inadequate keratinized tissue rather than other mucogingival abnormalities. Collectively, these findings suggest that baseline periodontal conditions may represent a more clinically relevant indicator of recession risk than the severity of pretreatment crowding alone. In the long term, gingival recession remains a multifactorial condition; however, thin periodontal phenotype, increasing age, and smoking history have been identified as risk factors, while mandibular central incisors appear to remain the most susceptible teeth [27].

Calibrated intraoral photographs were used to assess multiple clinical parameters in the present study both before and at the end of orthodontic treatment. This allowed the standardized evaluation of gingival recession, keratinized tissue width, and oral hygiene across the relatively large retrospective data. Photographic measurements have been used in previous orthodontic studies and provide a practical method for evaluating periodontal outcomes when direct clinical examination is not feasible [16,20]. However, this methodology, due to its retrospective nature, limited the ability to assess certain periodontal characteristics, particularly gingival thickness, periodontal phenotype, and probing depth, which have been suggested as potential risk factors for recession development.

In addition, certain orthodontic variables, including lower incisor proclination, arch expansion, and direction of tooth movement, were not consistently available within the retrospective records and therefore could not be incorporated into the present analysis. The absence of these variables limits the ability to fully account for factors that may contribute to recession development independently of baseline crowding severity.

Potential sources of bias include the retrospective design and reliance on photograph-based measurements, which may be affected by image angulation, soft tissue positioning, and visibility of anatomical landmarks. Efforts were made to minimize measurement bias through standardized calibration procedures and repeated measurements performed by a calibrated examiner. Although multivariable adjustment was performed for available confounding variables, residual confounding cannot be completely excluded. Additionally, despite merging the crowding categories into three for multivariable analysis, smaller subgroup sizes may have limited the ability to detect weak associations between crowding severity and recession development. Therefore, the findings should be interpreted within the context of these methodological limitations and unmeasured orthodontic and periodontal factors.

## 5. Conclusions

Overall, the present results indicate that pretreatment mandibular anterior crowding was not associated with post-treatment gingival recession in this cohort. In contrast, pretreatment mucogingival deformities were independently associated with recession development. This supports careful periodontal evaluation and continued periodontal care during orthodontic therapy Future longitudinal studies incorporating periodontal phenotype, orthodontic tooth movement variables, and long-term follow-up are needed to further clarify factors associated with gingival recession development following orthodontic treatment.

## Figures and Tables

**Table 1:** Demographic characteristics of the study population.

Characteristics	N	Overall (*n* = 269)
Sex	269	
Male		120 (45%)
Female		149 (55%)
Pre-treatment age ^1^	269	14
Treatment duration (months) ^1^	269	25 (22.28)
Type of malocclusion	269	
Angle’s Class 1		175 (65%)
Angle’s Class 2		79 (29%)
Angle’s Class 3		15 (5.6%)
Little’s irregularity index groups	269	
Perfect alignment		57 (21%)
Minimal irregularity		95 (35%)
Moderate irregularity		77 (29%)
Severe irregularity		32 (12%)
Very severe irregularity		8 (3.0%)
Degree of crowding (mm)		3.00 (1.20, 5.10)
Arch-tooth size discrepancy (mm)	269	−3.5 (−5, −1.5)
Spee correction (mm)	269	−2.5 (−3.5, −2.00)
Net (mm)	269	−6 (−8.00, −4.00)
Crowding categories	269	
No crowding		4 (1.5%)
Mild		42 (16%)
Moderate		135 (50%)
Severe		69 (26%)
Very severe		19 (7.1%)
Oral hygiene prior to orthodontic treatment	269	
Good		178 (66%)
Fair		91 (34%)
Poor		0 (0%)

Data are presented as n (%) or median (interquartile range [IQR]), unless otherwise indicated. SD, standard deviation; IQR, interquartile range; KT, keratinized tissue.

**Table 2:** Pretreatment clinical parameters.

Parameter	N (%)						*p*-Value
Tooth number	33	32	31	41	42	43	
Pretreatment lack of KT	138 (51%)	103 (38%)	138 (51%)	146 (54%)	114 (42%)	148 (55%)	<0.001 *
Pretreatment recession	7 (2.6%)	4 (1.9%)	5 (1.9%)	6 (2.2%)	2 (0.7%)	3 (1.1%)	0.6

KT, keratinized tissue. Data are presented as n (%). *p*-values were calculated using Pearson’s chi-square test. * Significant difference in prevalence of lack of keratinized tissue among tooth types (*p* < 0.05).

**Table 3:** Distribution of recession prevalence at the end of orthodontic treatment.

**Parameter**	**N (%)**						***p*** **-Value**
Recession in any tooth	76 (4.7%)						
Tooth number	33	32	31	41	42	43	
Recession at the end of treatment	14 (5.2%) ^‡^	4 (1.5%) ^α,^***	24 (8.9%) ^‡^	20 (7.4%) ^‡^	2 (0.7%) ^α,^***	12 (4.5%) ^‡^	<0.001

Data are presented as n (%). *p*-values were calculated using Pearson’s chi-square test. Pairwise comparisons with Bonferroni adjustment were performed when applicable. *** Significant difference with teeth 33 and 43; ^‡^ significant difference with teeth 32 and 42; ^α^ significant difference with teeth 31 and 41 (*p* < 0.001).

**Table 4:** Comparison of characteristics between patients with overall gingival recession and those without recession.

**Characteristic**	**No Overall Recession (n = 221)**	**Overall Recession (n = 48)**	***p*** **-Value**
Pre-treatment age (years)			0.9
12	37 (17%)	11 (23%)	
13	44 (20%)	7 (15%)	
14	52 (24%)	14 (29%)	
15	40 (18%)	7 (15%)	
16	23 (10%)	4 (8.3%)	
17	18 (8.1%)	3 (6.3%)	
18	7 (3.2%)	2 (4.2%)	
Type of malocclusion			0.2
Angle’s Class 1	148 (67%)	27 (56%)	
Angle’s Class 2	60 (27%)	19 (40%)	
Angle’s Class 3	13 (5.9%)	2 (4.2)	
Little irregularity index			0.7
Perfect	48 (22%)	9 (19%)	
Mild	80 (36%)	15 (31%)	
Moderate	63 (29%)	14 (29%)	
Severe	24 (11%)	8 (17%)	
Very severe	6 (2.7%)	2 (4.2%)	
Degree of crowding (mm)			
Crowding categories			0.5
No crowding	4 (1.8%)	0 (0%)	
Mild	35 (16%)	7 (15%)	
Moderate	113 (51%)	22 (46%)	
Severe	56 (25%)	13 (27%)	
Very severe	13 (5.9%)	6 (13%)	
Poor	0 (0%)	0 (0%)	
Pre-treatment OH			0.064
Good	152 (69%)	26 (54%)	
Fair	69 (31%)	22 (46%)	
Poor	0 (0%)	0 (0%)	
Post-treatment OH			0.043 *
Good	131 (59%)	23 (48%)	
Fair	78 (35%)	25 (52%)	
Poor	12 (5.4%)	0 (0%)	

* Data are presented as n (%) or median (IQR). *p*-values were calculated using Pearson’s chi-square test, Fisher’s exact test, or Wilcoxon rank-sum test, as appropriate. Statistically significant at *p* < 0.05. OH, oral hygiene; IQR, interquartile range.

**Table 5:** Multivariable logistic regression analysis for factors associated with post-treatment gingival recession.

**Variable**	**Adjusted OR**	**95% CI**	***p*** **-Value**
Crowding severity group (ref: Perfect/Mild)			
Moderate vs. Perfect/Mild	1.03	0.49–2.15	0.937
Severe/Very Severe vs. Perfect/Mild	1.43	0.61–3.38	0.411
Age (per 1-year increase)	0.98	0.80–1.19	0.803
Sex: Female vs. Male	1.17	0.62–2.22	0.631
Post-treatment oral hygiene: Fair/Poor vs. Good	1.45	0.77–2.72	0.251
Pretreatment mucogingival deformity: Present vs. Absent	2.57	1.13–5.83	0.024

aOR, adjusted odds ratio; CI, confidence interval. The dependent variable was the presence versus absence of post-treatment gingival recession. The model included age, sex, crowding severity group, post-treatment oral hygiene, and pretreatment mucogingival deformity. A total of 269 patients were included, with 48 recession events. No evidence of multicollinearity was observed.

## Data Availability

The datasets generated and/or analyzed during the current study in the form of Excel tables are available from the corresponding author on reasonable request.
